# Integrating Genomic Data into Public Health Surveillance for Multidrug-Resistant Organisms, Washington, USA

**DOI:** 10.3201/eid3113.241227

**Published:** 2025-05

**Authors:** Laura Marcela Torres, Jared Johnson, Audrey Valentine, Audrey Brezak, Emily C. Schneider, Marisa D’Angeli, Jennifer Morgan, Claire Brostrom-Smith, Chi N. Hua, Michael Tran, Darren Lucas, Joenice Gonzalez De Leon, Drew MacKellar, Philip Dykema, Kelly J. Kauber, Allison Black

**Affiliations:** Washington State Department of Health, Shoreline, Washington, USA (L.M. Torres, J. Johnson, A. Valentine, A. Brezak, E.C. Schneider, M. D’Angeli, C.N. Hua, M. Tran, D. Lucas, J. Gonzalez De Leon, D. Mackellar, P. Dykema, K.J. Kauber, A. Black); Public Health Seattle and King County, Seattle, Washington, USA (J. Morgan, C. Brostrom-Smith)

**Keywords:** antimicrobial resistance, AMR, multidrug resistance, genomics, MDRO, AMR surveillance, bioinformatics, epidemiology, bacteria, Klebsiella pneumoniae, Pseudomonas aeruginosa, Acinetobacter baumannii, bacterial outbreak, genomic cluster, Washington, United States

## Abstract

Mitigating antimicrobial resistance (AMR) is a public health priority to preserve antimicrobial treatment options. The Washington State Department of Health in Washington, USA, piloted a process to leverage longitudinal genomic surveillance on the basis of whole-genome sequencing (WGS) and a genomics-first cluster definition to enhance AMR surveillance. Here, we outline the approach to collaborative surveillance and describe the pilot using 6 carbapenemase-producing organism outbreaks of 3 species: *Pseudomonas aeruginosa*, *Acinetobacter baumannii*, and *Klebsiella pneumoniae.* We also highlight how we applied the approach to an emerging outbreak. We found that genomic and epidemiologic data define highly congruent outbreaks. By layering genomic and epidemiologic data, we refined linkage hypotheses and addressed gaps in traditional epidemiologic surveillance. With the accessibility of WGS, public health agencies must leverage new approaches to modernize surveillance for communicable diseases.

Multidrug resistance threatens modern medicine and public health by limiting our ability to effectively treat serious infections (*1*). Accordingly, reducing and preventing antimicrobial resistance (AMR) is a high priority. Of particular concern are carbapenemase-producing organisms (CPOs), a subset of multidrug-resistant organisms (MDROs) that are resistant to carbapenems—an important class of antibiotics typically reserved as a last resort—and associated with high mortality rates (*2*). CPOs can transfer their resistance genes via mobile genetic elements, like plasmids, across multiple species, contributing to the proliferation of AMR (*3**,**4*). CPOs and plasmids carrying carbapenemase genes have the potential to make all current antimicrobial drugs ineffective; as such, public health prioritizes surveillance and containment of AMR. Comprehensive strategies critical to mitigate AMR, including antimicrobial stewardship; prompt, accurate diagnosis, and treatment; and infection prevention and control to limit transmission, depend on AMR surveillance data (*5**–**7*). Recognizing those needs, global and national public health agencies advocate for robust AMR surveillance systems providing timely, high-quality data to inform global, regional, and local containment strategies (*5**,**8*). By incorporating complementary data sources, robust AMR surveillance systems enable early warning of pathogen emergence, enhance monitoring of epidemiologic trends, improve detection of outbreaks, and deepen understanding of transmission events.

AMR surveillance and cluster investigations rely on epidemiology of person, place, and time, coupled with the genetic and phenotypic characteristics of suspected pathogens. Whole-genome sequencing (WGS) has become a standard method for determining genetic characteristics of pathogens because it enables more comprehensive AMR gene detection compared with traditional PCR-based methods. WGS also enables full-genome comparisons between isolates through core-genome single-nucleotide polymorphism (SNP) analysis. This wider view offers superior resolution over traditional methods that only consider a fraction of the genome, such as multilocus sequence typing (MLST). That resolution reduces misclassification and other biases when making inferences about transmission events (*7*,*9*) and improves our ability to differentiate related and unrelated cases (*10*). Taken together, WGS data enable us to detect MDRO clusters earlier and deploy infection control interventions more quickly (*11*,*12*), detect genes associated with AMR, determine whether resistance is due to chromosomal mutations or to mobile resistance genes (*7*,*13*,*14*), and build genomic datasets that provide context for prospective analyses (*11*).

Given the potential of WGS to advance routine AMR surveillance, we developed and integrated a genomics-first approach into our AMR surveillance system at the Washington State Department of Health. Select MDROs, including CPOs, *Candida auris*, and vancomycin-resistant *Staphylococcus aureus*, are the focus of MDRO surveillance in Washington. Within this system, MDRO sequencing data, generated by Washington Public Health Laboratory (WAPHL) via the Centers for Disease Control and Prevention (CDC)–funded Antibiotic Resistance Laboratory Network (ARLN) testing activities, are ingested into recombination-aware bioinformatics pipelines to identify genomic relationships. Then, data are passed through a workflow that sources and combines surveillance and genomic data. Central to that approach, we established communication and reporting protocols to foster collaborative discussion between laboratory and epidemiology programs about inferences derived from the different data sources. We piloted the approach on 6 historical MDRO outbreaks to explore congruence between genomically and epidemiologically defined clusters and to assess the additive effect of integrating genomic information. Here, we present the results of the pilot and show how to use the integrated surveillance system to support MDRO outbreak investigations prospectively.

## Methods

WAPHL, the Multidrug-Resistant Organism Program (MDROP), and the Molecular Epidemiology Program (MEP) teams at the Washington State Department of Health analyzed 6 known MDRO outbreaks across 3 species, *A. baumannii*, *P. aeruginosa*, and *K. pneumoniae*, and multiple health facilities (Table 1). The outbreaks were identified through laboratory detection of targeted CPOs by clinical laboratories or WAPHL through the ARLN; methods are summarized on CDC’s ARLN Testing Web site (*15*). Cases were identified by detection of a carbapenemase in clinical isolates and through colonization screening performed for MDRO containment response or admission screening. Using epidemiologic investigation methods, MDROP and local health jurisdictions identified linked cases.

**Table 1:** Overview of 6 outbreaks of multidrug-resistant organism outbreaks, Washington, USA*

Outbreak ID	Pathogen	No. linked cases	No. health facilities
1	*Pseudomonas aeruginosa *	3	1
2	*Acinetobacter baumannii*	5	1
3	*A. baumannii*	6	1
4	*A. baumannii*	6	1
5	*A. baumannii*	5	5
6	*Klebsiella pneumoniae *	5	1

*We determined linkages between cases by epidemiologic evidence. ID, identification.

### Epidemiologic Data

CPOs are reportable in Washington; public health staff investigate all CPOs in partnership with affected healthcare facilities and manage patient screening among epidemiologically linked healthcare contacts. MDROP partners with local health jurisdictions to perform longitudinal surveillance using an Antimicrobial Resistance Information Exchange (ARIE), investigate potential clusters, perform containment responses, and document reported outbreaks. For this pilot, MDROP provided MEP a master list for each of the 6 outbreaks, including epidemiologic information about index cases, facility admissions, known epidemiologic linkages, and isolate identifiers to link case and sequencing data.

### Sequencing and Genomic Analysis

We performed WGS using DNA extracted with the MagNA Pure 96 Small Volume Kit on an MP96 system (both Roche, https://ww.roche.com) from bacterial cultures grown on blood agar (Thermo Fisher Scientific, https://www.thermofisher.com) for 24 hours at 35–37°C. We prepared paired-end DNA libraries using the Illumina DNA Prep kit with Nextera DNA CD indexes sequenced on a MiSeq System (all Illumina, https://www.illumina.com) using the 2 × 250 bp (500-cycle) v2 kit. We used the CDC PHoeNIx pipeline (https://zenodo.org/record/8147510) to perform general bacterial analysis, including quality control, de novo assembly, taxonomic classification, and AMR gene detection. We repeated sequencing for samples with <40× average read depth, <1 Mb genome size, >500 assembly scaffolds, or >2.58 assembly ratio SD (Appendix 1 Table 1). PHoeNIx outputs feed into the WAPHL BigBacter pipeline (https://github.com/DOH-JDJ0303/bigbacter-nf), which enables bacterial genomic surveillance by performing phylogenetic analysis and differentiating clusters of closely related bacteria that are maintained in a personalized database. We clustered samples genomically using PopPUNK version 2.6.0 as described (*16*) and calculated accessory distances and core SNPs within each genomic cluster using the PopPUNK sketchlib functions and Snippy version 4.6.0 (https://github.com/tseemann/snippy). We identified and masked recombinant regions in the Snippy output using Gubbins version 3.3.1 as described (*17*). We generated phylogenetic trees and distance matrices using IQTREE2 version 2.2.2.6 as described (*18*) and custom scripts in R (The R Project for Statistical Computing, https://www.r-project.org) and Bash (Free Software Foundation, Inc., https://www.gnu.org/software/bash).

We linked the BigBacter genomic outputs to metadata attributes queried from our laboratory information and surveillance systems, enabling joint analysis and visualization in R and Nextstrain Auspice (*19*). We used the phylogenetic trees, SNP matrices, and BigBacter’s cluster designation to identify genomic clusters. To explore congruence between genomic clusters and epidemiologically defined clusters in our pilot, we identified the subset of genomic clusters that grouped cases associated with 6 outbreaks defined by MDROP. Then, we looked at the union of all sequenced samples in relevant genomic clusters (n = 43) and all cases identified as part of the 6 epidemiologically defined outbreaks (n = 36). We defined samples as follows: genomically linked only, meaning that the sequenced sample grouped in a relevant genomic cluster and either the core genome sequences were closely related (<10 SNPs) or a larger SNP distance could be explained by differences in sample collection dates; epidemiologically linked only, meaning that MDROP had linked a case to an outbreak, but that the sequence did not meet the genomically linked definition; or epidemiologically and genomically linked, meaning that both MDROP epidemiologists’ assessment and sequencing data grouped the case as part of the relevant outbreak. MEP, WAPHL, and MDROP met to discuss the findings. Communication between our programs helped address perceived utility of routine genomic analyses and enabled us to develop processes for ongoing data production, analytics, interpretation, and cross-program communication.

## Results

### Cluster Detection Using a Genomics-First Approach 

**Figure 1:**
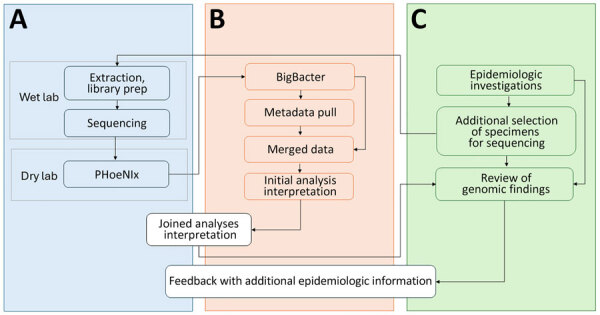
Data flow and cross-team communication channels for our system for integrating genomic data into public health surveillance for multidrug-resistant organisms, Washington, USA. This diagram shows how a sample, then data, move through our integrated surveillance program. Tasks that are handled jointly across programs are highlighted in white. A) Tasks conducted by Washington Public Health Laboratory. B) Tasks conducted by Molecular Epidemiology Program. C) Tasks conducted by Multidrug-Resistant Organism Program and local health jurisdictions.

**Figure 2:**
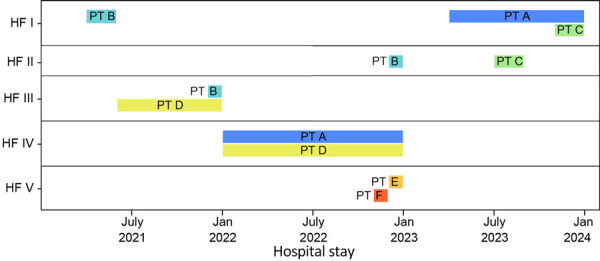
Timeline showing overlap of patients with *Klebsiella pneumoniae* carbapenemase–producing *K. pneumoniae* infection in healthcare facilities in Washington, USA, as part of study of integrating genomic data into public health surveillance for multidrug-resistant organisms. PTs A, B, and C stayed in HF I. PT A might have overlapped with PT C in HF I in 2023; PT B stayed in HF I in 2021. PTs B and C both stayed in HF II but at different times, in 2022 and in 2023. PT D stayed at HF III in 2021, where an overlap with PT B might have occurred, and in 2022, PT D might have overlapped with PT A in HF IV. PTs E and F who had stayed in HF V could also be related to this outbreak. HF, health facility; PT, patient.

**Figure 3:**
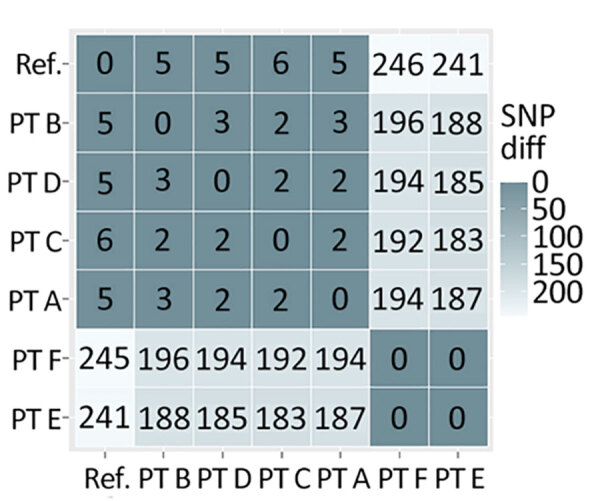
SNP matrix showing number of polymorphic sites observed when making pairwise comparisons between the core genome of the sequences in a cluster of *Klebsiella pneumoniae* carbapenemase–producing *K. pneumoniae* isolates as part of study of integrating genomic data into public health surveillance for multidrug-resistant organisms, Washington, USA. Dark gray represents lower SNP distances and light gray larger SNP distances. Diff, difference; PT, patient; ref., reference; SNP, single-nucleotide polymorphism.

**Figure 4:**
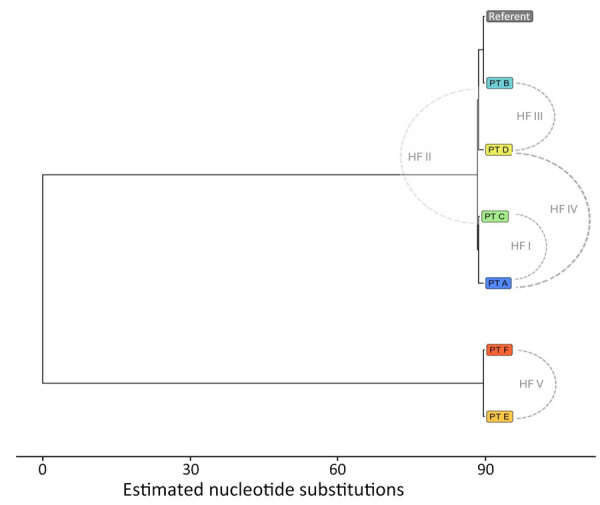
Maximum-likelihood phylogenetic tree of sequences from patients with *Klebsiella pneumoniae*–producing *K. pneumoniae* infection as part of study of integrating genomic data into public health surveillance for multidrug-resistant organisms, Washington, USA. Five patients (A–E) are shown, and relevant HFs are noted. HF, health facility; PT, patient.

**Figure 5:**
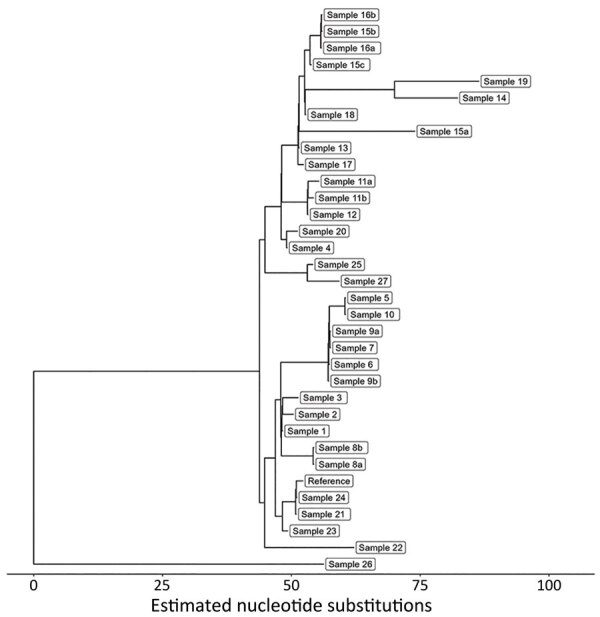
Maximum-likelihood phylogenetic tree showing relationships among 33 carbapenemase-producing *Acinetobacter baumannii* isolates with the OXA-235–like carbapenemase gene as part of study of integrating genomic data into public health surveillance for multidrug-resistant organisms, Washington, USA.

**Figure 6:**
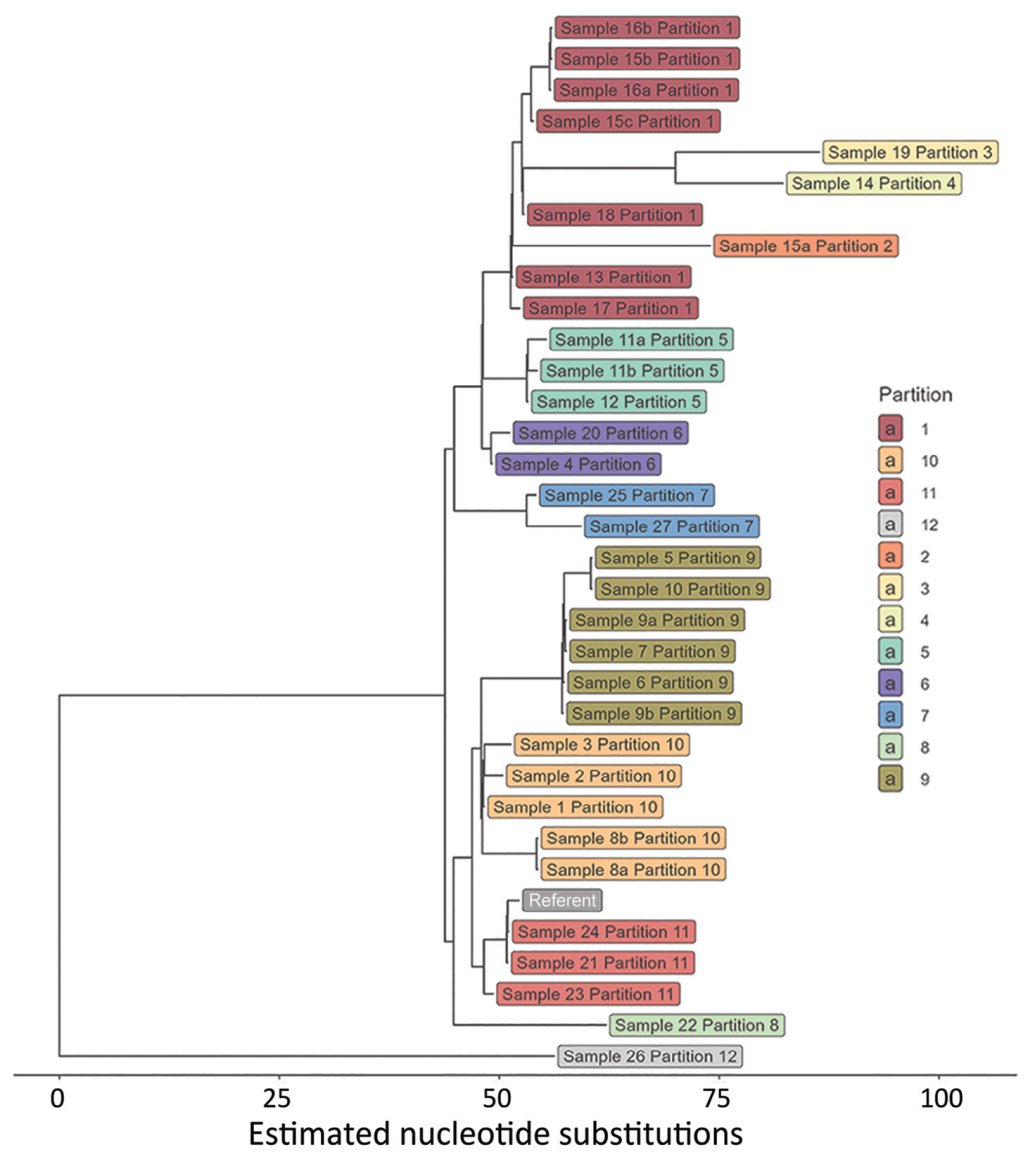
Maximum-likelihood phylogenetic tree showing partitions of 33 carbapenemase-producing *Acinetobacter baumannii* isolates with the OXA-235–like carbapenemase gene as part of study of integrating genomic data into public health surveillance for multidrug-resistant organisms, Washington, USA. Colors indicate 12 partitions demarcating sequences separated by <10 SNPs. Seven of the partitions contain multiple sequences and 5 (2, 3, 4, 8, and 12) contain 1 sequence.

To pilot integrated surveillance, we evaluated whether genomic data and epidemiologic investigations grouped the same cases for 6 known, epidemiologically defined outbreaks. We analyzed 221 sequences of *P. aeruginosa*, *A. baumannii*, and *K. pneumoniae*, collected during December 2017–May 2024; those sequences grouped into 48 genomic clusters. Six of the genomic clusters were largely concordant with the 6 epidemiologically defined outbreaks (n = 36 cases). The 6 genomic clusters grouped 42 sequences, of which 32 were classified as epidemiologically and genomically linked (Table 2; Appendix 1 Figures 1–6). One epidemiologically linked case grouped into a seventh genomic cluster with no other linked cases, indicating that genomic data did not support the linkage. Although BigBacter groups related sequences, pairwise genetic divergence within a cluster can still exceed the SNP distance threshold we use to define genomic linkage. Indeed, 4 epidemiologically linked cases grouped into outbreak-related genomic clusters but were not considered genomically linked because they diverged from other sequenced cases by 14–56 SNPs; that distance could not be explained by differences in sample collection dates (Table 2; Appendix 1 Figures 4, 6). Six sequences grouped into relevant genomic clusters with minimally divergent core genome sequences, but those cases had not been linked to the outbreaks through epidemiologic information; the cases were genomically linked only (Table 2; Appendix 1 Figures 1, 4, 5). Our findings show general concordance between epidemiologic and genomic clusters and demonstrate instances where genomic data may refine cluster definitions.

**Table 2:** Results of pilot study of genomic and epidemiologic surveillance of outbreaks of multidrug-resistant organism infections, Washington, USA*

Outbreak ID	Pathogen	No. health facilities	No. cases, n = 36	No. isolates sequenced, n = 43	Epidemiologically linked only, n = 5	Epidemiologically and genomically linked, n = 32	Genomically linked only, n = 6
1	*Pseudomonas aeruginosa*	1	5	8†	0	6	2
2	*Acinetobacter baumannii*	1	5	6‡	0	6	0
3	*A. baumannii*	1	6	6	0	6	0
4	*A. baumannii*	1	7	10†	3	6	1
5	*A.baumannii*	5	8	8	0	5	3
6	*Klebsiella pneumoniae*	1	5	5	2§	3	0

*ID, identification. †One case had 3 isolates sequenced and 1 had 2 isolates sequenced. ‡One case had 2 isolates sequenced. §Sample 5 was placed into a separate genomic cluster due to relatively large pairwise genetic differences between this isolate and the remaining outbreak 6 isolates, as determined by PopPUNK (*16*).

### Development of Standard Integrated Genomic Epidemiology Reports

We sought to develop mechanisms to jointly analyze genomic and epidemiologic data and communicate across teams about the inferences. MEP, MDROP, and WAPHL discussed the pilot study findings, including the utility and limitations of genomic analyses, and collectively designed a new data and communication workflow. The workflow required us to bridge siloed data sources (Figure 1); to do so, we programmatically ingest laboratory identifiers and query the surveillance database. Working with MDROP, we determined which epidemiologic information are most important for contextualizing genomic information (e.g., submitter facility name, submitter county, collection date, etc.). We source, format, and export this information as a metadata file that can be overlaid onto phylogenetic trees.

MEP and WAPHL iteratively refined the information included in the reports to meet MDROP’s needs. The current version of the report includes 3 components. The first component is an automated R markdown-based report that parses the BigBacter output and metadata to summarize key information, such as the total number of sequences per genomic cluster, number of new sequences added to previously identified clusters, submitting health facilities and counties, and sequences with close or intermediate genomic linkage (Appendix 2). The second component is a narrative interpretation of the genomic data written by MEP epidemiologists; that component alerts MDROP epidemiologists to transmission dynamics consistent with the genomic data, such as detection of new introductions or ongoing transmission of an outbreak. The final component of the report is a Microreact (*20*) dashboard, where we share interactive multipanel figures including SNP distance matrices and phylogenetic trees; this type of reporting is a standard feature of Washington’s AMR surveillance. Among other outcomes, the approach has improved our understanding of *K. pneumoniae* transmission within a multifacility outbreak and helped us ascertain linkages between carbapenemase-producing *A. baumannii* (CRAB) cases that were previously unknown. 

### Differentiation of Outbreak and Nonoutbreak Samples Using Genomic Data

In a prospective analysis of a* Klebsiella pneumoniae* carbapenemase–producing *K. pneumoniae* outbreak involving multiple healthcare facilities, epidemiologic investigation data alone could not clarify how transmission had occurred; recent healthcare during the exposure period involved multiple cases, some with overlapping healthcare stays (Figure 2). Integrating genomic and case-level data helped us refine relationships between cases and formulate a hypothesis for how cases were connected across facilities. MEP and WAPHL reported that sequences from patients A, B, and C were closely related (2–3 SNPs) (Figure 3). MDROP confirmed epidemiologic linkages among some of those patients (Figures 2, 3), but a common link was missing. MDROP hypothesized that patients D, E, or F could be the missing link and requested a review of their sequencing results, pending sequencing for patient D. MDROP’s reasoning was that patient D might have overlapped with patients A and B. Sequencing revealed that patients E and F had identical core-genome sequences but diverged greatly from the other sequenced cases (Figure 4). MDROP confirmed an epidemiologic link between patients E and F, noting they received care at the same facility and shared staff. The genomic and epidemiologic information helped confirm these patients were connected to each other but not related to the outbreak in question. The sequence from patient D, however, was genomically linked (2–3 SNPs) to sequences from patients A, B, and C (Figure 3). The close genomic distances and the overlap in healthcare stays with patients A and B supported the hypothesis that patient D was one of the missing links. Patient C’s relationship to the outbreak remains unclear; patient C tested positive upon admission but reported no healthcare encounters before August 2023. Despite that remaining question, genomic analyses helped confirm 1 missing link, excluded 2 patients from this outbreak, and revealed that the outbreak was larger than originally thought.

### Genomic Data Linking Historical Carbapenemase-Producing *A. baumannii* Cases

We assessed the congruence between epidemiologic surveillance data and genomic clustering for a retrospective set of CRAB isolates with the OXA-235–like carbapenemase gene. Two outbreaks were known to MDROP at healthcare facilities I and IV. First, we reviewed all 33 sequenced CRAB OXA-235 isolates representing 27 cases collected during August 2019–December 2023. We compiled healthcare encounters for cases from MDROP’s linelist and ARIE and matched 137 admissions across 29 facilities from July 2020–May 2024. We visualized genomic analyses and epidemiologic data using vistime and ggtree (https://shosaco.github.io/vistime) (*21*) in R.

**Figure 7:**
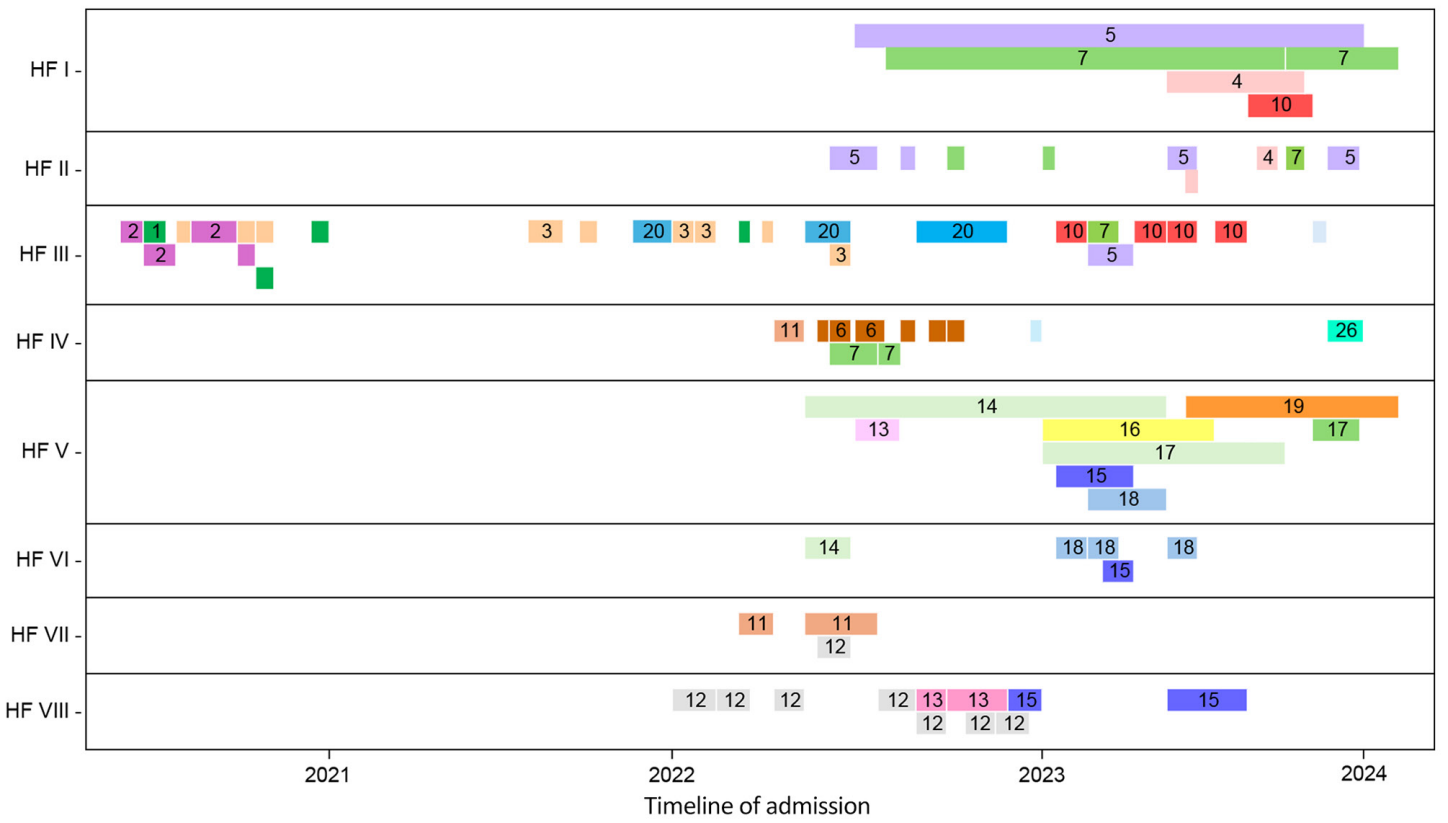
Healthcare encounters at facilities of interest among carbapenemase-producing *Acinetobacter baumannii* OXA-235 cases as part of study of integrating genomic data into public health surveillance for multidrug-resistant organisms, Washington, USA. Six cases (14, 15, 16, 17, 18, and 19) are linked to a screening event at HF V, and 3 cases (5, 7, and 10) are linked to a screening event at HF I. Cases cannot be in 2 or more health facilities simultaneously. However, we only have access to admission and discharge dates; therefore, the figure may show some cases in multiple locations at the same time if transfers occurred without an associated admission and discharge. HF, health facility.

We used PopPUNK (*16*) for genomic clustering; all 33 isolates were assigned to the same genomic cluster (Figure 5). The cluster had a maximum pairwise divergence of 119 SNPs. To identify closer genetic relationships indicative of clonal transmission, we used BigBacter to partition the cluster into groups of sequences separated by ≤10 SNPs (*22**,**23*), resulting in 12 partitions (Figure 6). Seven partitions contained multiple sequences. We defined sequences within a partition as genomically linked to each other. In this analysis, MDROP defined epidemiologic linkage between cases as temporally overlapping visits at the same healthcare facility. We considered 8 facilities that had cases with overlapping visits to be facilities of interest (Figure 7). We categorized cases that were epidemiologically linked and belonged to the same genomic partition as epidemiologically and genomically linked. We evaluated concordance between genomic and epidemiologic data by categorizing sequences from the 7 partitions as epidemiologically and genomically linked, epidemiologically linked only, or genomically linked only. Four partitions (1, 5, 9, and 10) included 21 sequences; we considered 17 of those epidemiologically and genomically linked and 4 genomically linked only. We classified the sequences in the remaining 3 multisequence partitions (6, 7, and 11) as genomically linked only; partition 6 contained 2 sequences from cases that were not epidemiologically linked, and sequences in partitions 7 and 11 were from cases missing epidemiologic data (Appendix 1 Table 1). Five partitions (2, 3, 4, 8, and 12) contained only 1 sequence and thus had no evidence of genomic linkage. Of those 5 sequences, we considered 3 epidemiologically linked (Appendix 1 Table 2); 2 sequences lacked epidemiologic data.

Our results highlight the consistency that genomically and epidemiologically defined clusters can have, as well as how our definition for epidemiologic linkage may lack sensitivity and specificity. Indeed, detailed retrospective case review prompted by genomic linkages described by our analysis yielded 10 epidemiologic links unknown to MDROP.

## Discussion

Here, we describe our approach to integrating genomics into our AMR surveillance system and transitioning from a pilot assessment to a repeatable workflow. Integrating genomic data into AMR surveillance has helped us identify additional outbreak cases, sensitively classify outbreak or nonoutbreak cases, and confirm hypothesized linkages. Furthermore, we reduced silos between programs, fostering collective discussion to guide data interpretation and next steps. Building on this success, we now perform automated genomic cluster detection for all MDRO bacterial pathogens sequenced at WAPHL, and we plan to expand this approach to other surveillance programs.

Our approach has some notable benefits. First, our system characterizes genomic relationships using distance-based analysis of sequence data. Although national surveillance systems in the United States such as PulseNet (*24*) and TB GIMS (*25*) have transitioned from MLST, only a predefined set of loci within the core genome are considered, and the set of relevant loci cannot expand on an outbreak-by-outbreak basis. Although sequence types delineate whether sequences are nearly identical or not, they do not allow epidemiologists to directly estimate genetic distances between sequences. Second, BigBacter by default stores genomic cluster information in a running database, providing historical context when analyzing new sequences. This is one of the beneficial features of systems such as PulseNet, as it enables detection of reemerging outbreaks or strains (*26*), but to our knowledge such approaches are rarely implemented and maintained by a single state agency. Finally, our system mitigates the bias that can arise when sequencing is prompted solely by epidemiologic hypotheses. By sequencing MDRO detections regardless of outbreak status and identifying clusters given genetic relatedness only, we draft genomics-informed hypotheses independent of hypotheses derived from epidemiologic investigation data. When findings from both data streams are consistent, it strengthens our belief that we understand transmission within the cluster, whereas discrepancies prompt us to reinvestigate or evaluate gaps specific to each data source. This approach stands in contrast to targeted sequencing efforts where sequencing occurs only upon request, such as when surveillance epidemiologists have defined an outbreak.

Despite those benefits, our integrated AMR surveillance system has some limitations. Ideally, our system would include environmental and nonhuman isolates to clarify risk for zoonotic and environmental transmission of CPOs to humans (*13*,*14*). However, we lack access to those sample types, and our system’s slow turnaround time limits its utility. In our system, bacterial sequencing proceeds from cultured isolates, resulting in genomic analysis being shared ≈1 month after carbapenemase detection. By then, WGS only provides post hoc confirmation about links that have been already identified, rather than real-time information to inform infection control practices. Finally, WGS is expensive, which could make this program unsustainable in the absence of stable and appropriate funding.

Through our efforts to develop, test, and deploy an integrated AMR surveillance system, MDROP can leverage pathogen genomics for public health response. During active investigations, MDROP can intervene when genomic links are identified, guiding actions to improve infection control practices. Furthermore, by developing this system collectively, our system includes perspectives from surveillance epidemiology, molecular epidemiology, and bioinformatics and reduces silos between teams. Building on initial successes, we continue to refine this system to increase the timeliness of genomic inferences and identify best practices to engage local health jurisdictions.

Appendix 1 Additional information about integrating genomic data into public health surveillance for multidrug-resistant organisms, Washington, USA.

Appendix 2 Example of automated summary report in study of integrating genomic data into public health surveillance for multidrug-resistant organisms, Washington, USA.
