# Supplementary material for: Integrating Genomic Data into Public Health Surveillance for Multidrug-Resistant Organisms, Washington, USA
Source: Emerg Infect Dis. 2025 May;31(Suppl 1):S25–34. doi: 10.3201/eid3113.241227 (PMC12078550; doi:10.3201/eid3113.241227)
Supplement: Appendix 1 — Additional information about integrating genomic data into public health surveillance for multidrug-resistant organisms, Washington, USA. [file 24-1227-Techapp-s1.pdf]

Article DOI: https://doi.org/10.3201/eid3113.241227

EID cannot ensure accessibility for supplementary materials supplied by authors. Readers who have difficulty accessing supplementary content should contact the authors for assistance.

Integrating Genomic Data into Public Health Surveillance for Multidrug-Resistant Organisms, Washington, USA

Appendix 1

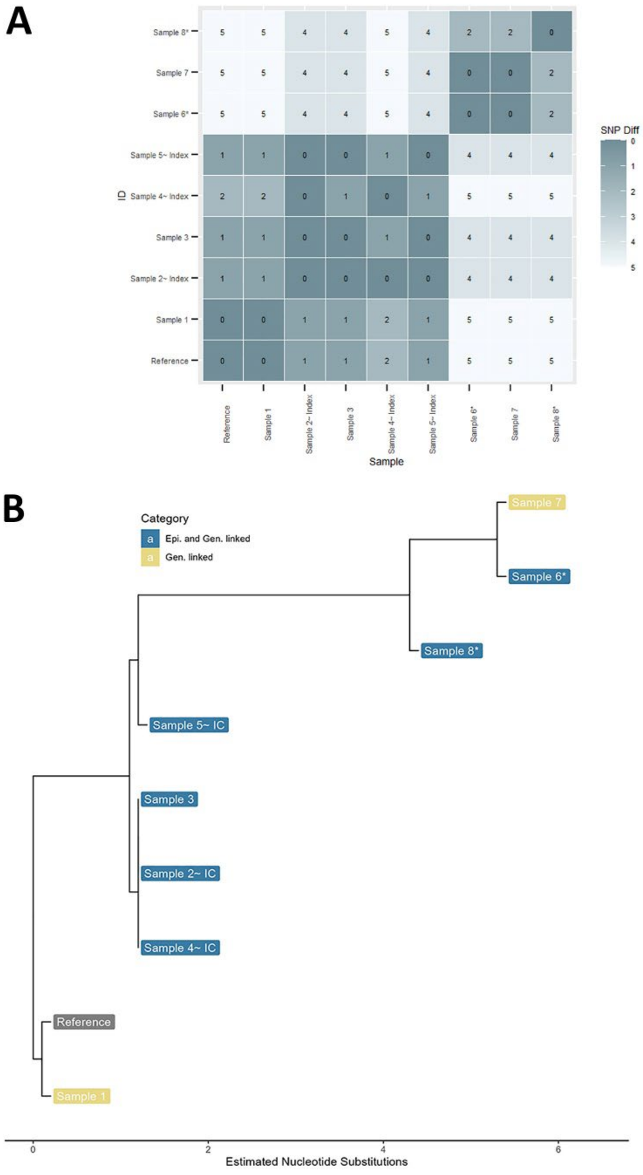

Appendix 1 Figure 1. A) *P. aeruginosa* SNP matrix and (B) maximum-likelihood phylogenetic tree summarize genomic and epidemiologic relationships from Outbreak 1. A) The SNP matrix shows the number of polymorphic sites observed when making pairwise comparisons between the core genome sequences in this cluster. The color-gradient goes from dark gray to light gray, with dark gray representing lower SNP values and light gray higher values. B) The phylogenetic tree shows genetic divergence between sequenced samples within this genomic cluster, where branch lengths correspond to core genome SNP distance between samples. Tips are labeled with anonymized case identifiers. Tips are colored blue if the individual was considered part of the outbreak by both epidemiologic and genomic analysis and yellow if the case is only considered linked to the outbreak due to sequence similarity to other outbreak cases. Estimated nucleotide substitutions define the eight sequences in this tree as closely related. Six cases in this cluster were confirmed as having epidemiologic linkages while 2 sequences that were genomically linked did not have epidemiologic linkages known to MDROP. Asterisk * indicates samples from the same case. ~IC, Samples from index case.

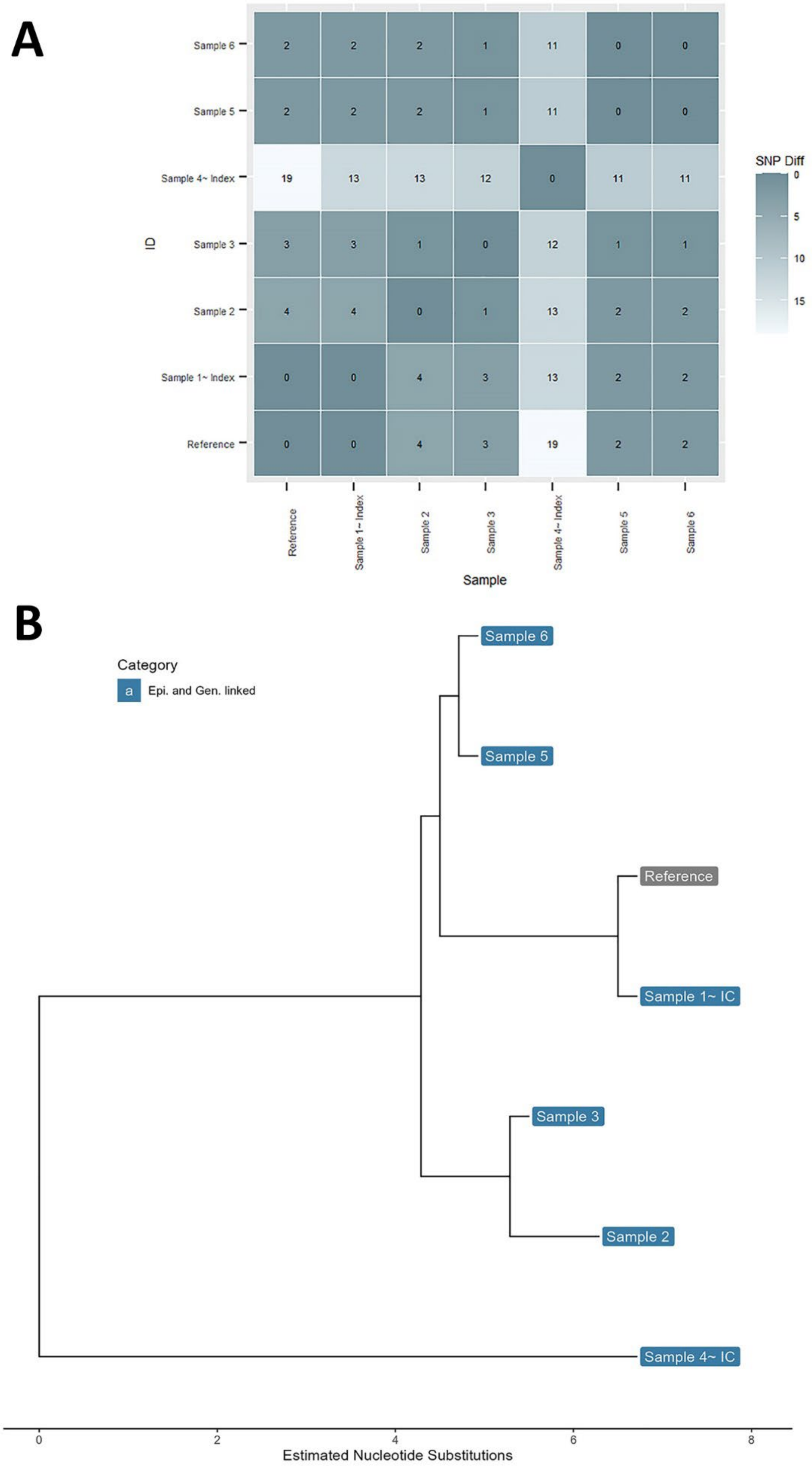
A
Sample 6
Sample 5
Sample 4~ Index
Sample 3
Sample 2
Sample 1~ Index
Reference
ID
Sample
SNP Diff
0
5
10
15
B
Category
a
Epi. and Gen. linked
Sample 6
Sample 5
Reference
Sample 1~ IC
Sample 3
Sample 2
Sample 4~ IC
0
2
4
6
8
Estimated Nucleotide Substitutions

Appendix 1 Figure 2. A) *A. baumannii* SNP matrix and (B) maximum-likelihood phylogenetic tree summarizing genomic and epidemiologic relationships from Outbreak 2. The SNP matrix and the maximum-likelihood phylogenetic tree are inferred, labeled, and colored as in Appendix Figure 1. Five out of 6 sequences within this cluster were closely genomically related (diverged by less than 10 SNPs). One sequence (Sample 4~IC) diverged from other sequences by 11–13 SNPs, including 13 SNPs from another sequence from the same patient 1 month earlier (Sample 1~IC). Despite the SNP distance greater than 10, we consider Sample 4~IC to be genomically linked with other cases because SNP distances of 11–13 SNPs are within the distance range that characterizes the within-host diversity of this patient. All samples within this cluster were linked to the same epidemiologically defined outbreak cluster, and MDROP later confirmed that the sequence from Sample 4 ~IC represented the outbreak's index case.

A

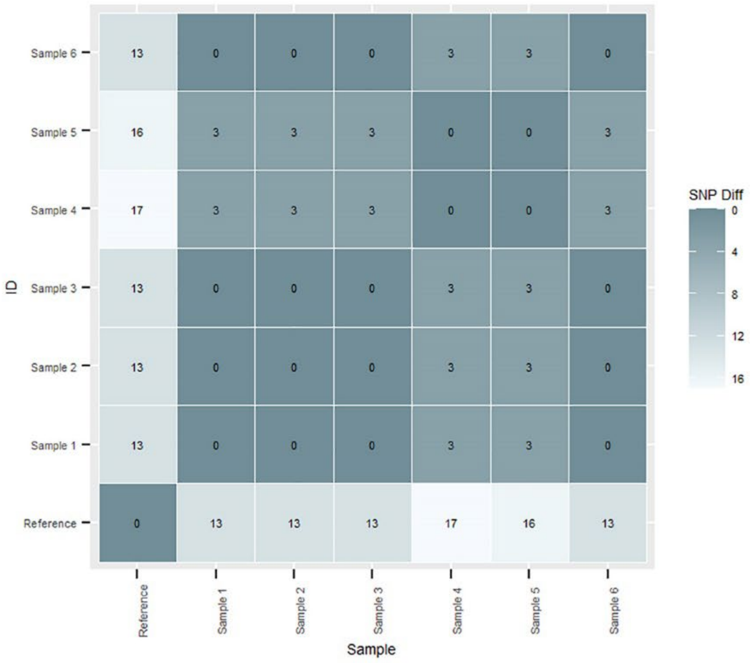
SNP Diff
0
4
8
12
16
ID
Sample
Reference
Sample 1
Sample 2
Sample 3
Sample 4
Sample 5
Sample 6

B

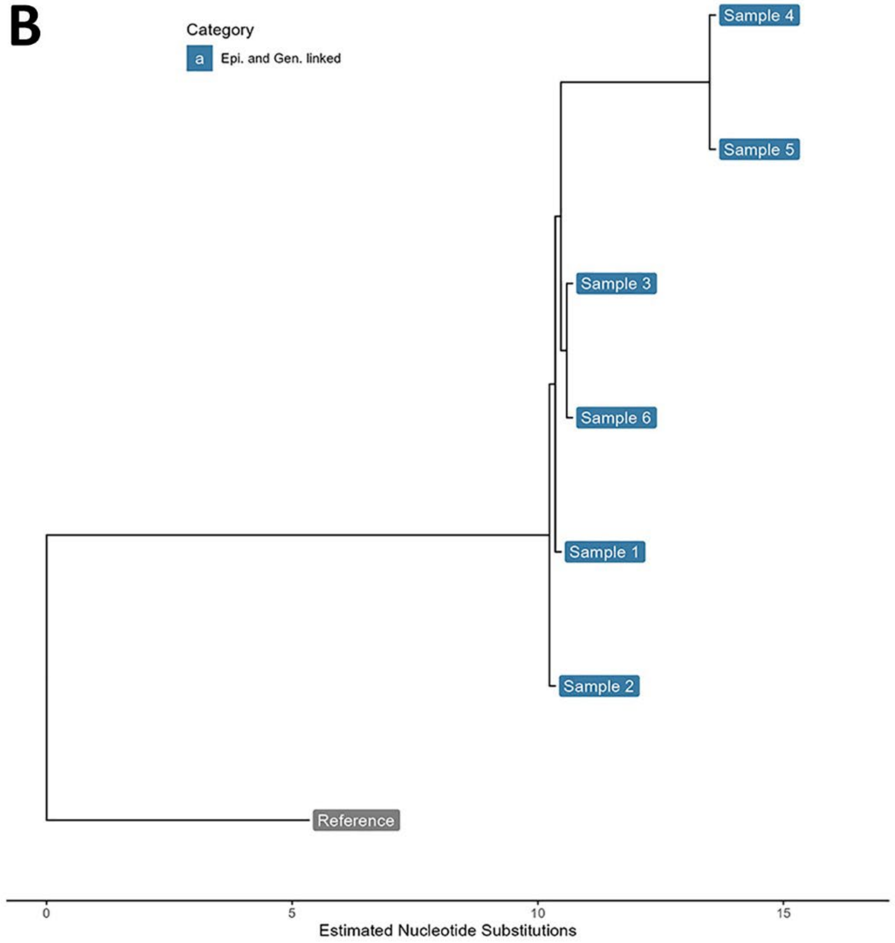
Category
a Epi. and Gen. linked
Sample 4
Sample 5
Sample 3
Sample 6
Sample 1
Sample 2
Reference
0
5
10
15
Estimated Nucleotide Substitutions

Appendix 1 Figure 3. A) *A. baumannii* SNP matrix and (B) maximum-likelihood phylogenetic tree summarize genomic and epidemiologic relationships from Outbreak 3. The SNP matrix and the phylogenetic tree are inferred, labeled, and colored as in Appendix Figure 1. All samples within this cluster were closely genomically related (diverged by less than 10 SNPs) and were sequenced from cases linked to the same outbreak during the epidemiologic investigation.

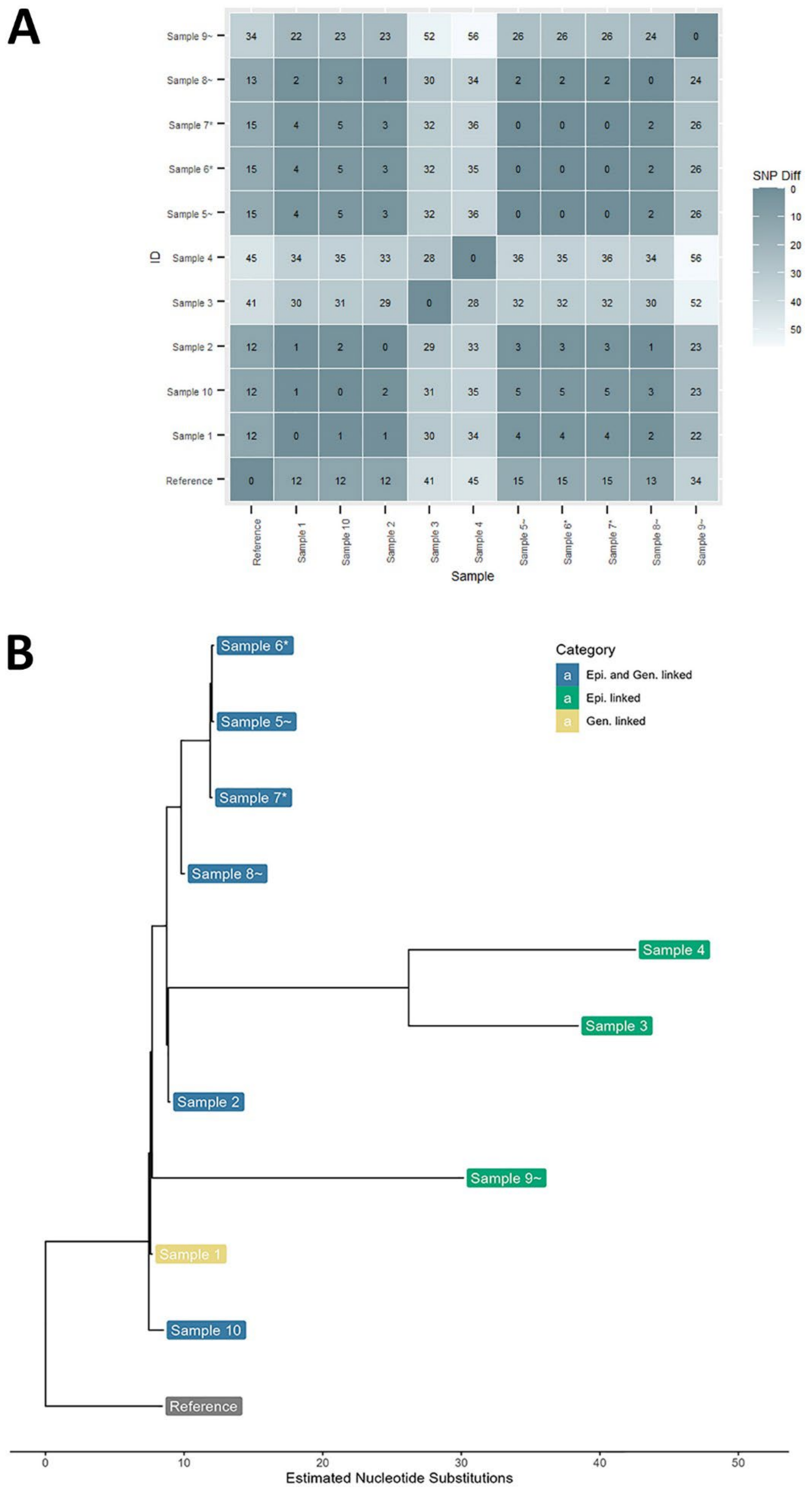
A
Sample 9~ 34 22 23 23 52 56 26 26 26 24 0
Sample 8~ 13 2 3 1 30 34 2 2 2 0 24
Sample 7* 15 4 5 3 32 36 0 0 0 2 26
Sample 6* 15 4 5 3 32 35 0 0 0 2 26
Sample 5~ 15 4 5 3 32 36 0 0 0 2 26
Sample 4 45 34 35 33 28 0 36 35 36 34 56
Sample 3 41 30 31 29 0 28 32 32 32 30 52
Sample 2 12 1 2 0 29 33 3 3 3 1 23
Sample 10 12 1 0 2 31 35 5 5 5 3 23
Sample 1 12 0 1 1 30 34 4 4 4 2 22
Reference 0 12 12 12 41 45 15 15 15 13 34
ID
Reference
Sample 1
Sample 10
Sample 2
Sample 3
Sample 4
Sample 5~
Sample 6*
Sample 7*
Sample 8~
Sample 9~
Sample
SNP Diff
0
10
20
30
40
50
B
Sample 6*
Sample 5~
Sample 7*
Sample 8~
Sample 4
Sample 3
Sample 2
Sample 9~
Sample 1
Sample 10
Reference
Category
Epi. and Gen. linked
Epi. linked
Gen. linked
0
10
20
30
40
50
Estimated Nucleotide Substitutions

Appendix 1 Figure 4. A) *A. baumannii* SNP matrix and (B) maximum-likelihood phylogenetic tree summarize genomic and epidemiologic relationships from Outbreak 4. The SNP matrix and the phylogenetic tree are inferred, labeled, and colored as in Appendix Figure 1. Within this cluster of ten sequences, nine were epidemiologically linked to a single outbreak using traditional investigation methods. Of these sequences, 6 sequences were closely related (less than 10 SNPs, tips in blue) and 3 sequences were epidemiologically linked only. One closely related sequence did not have epidemiologic linkages known to MDROP (Sample 1, tip in yellow). Asterisk * and ~ indicates samples from the same case.

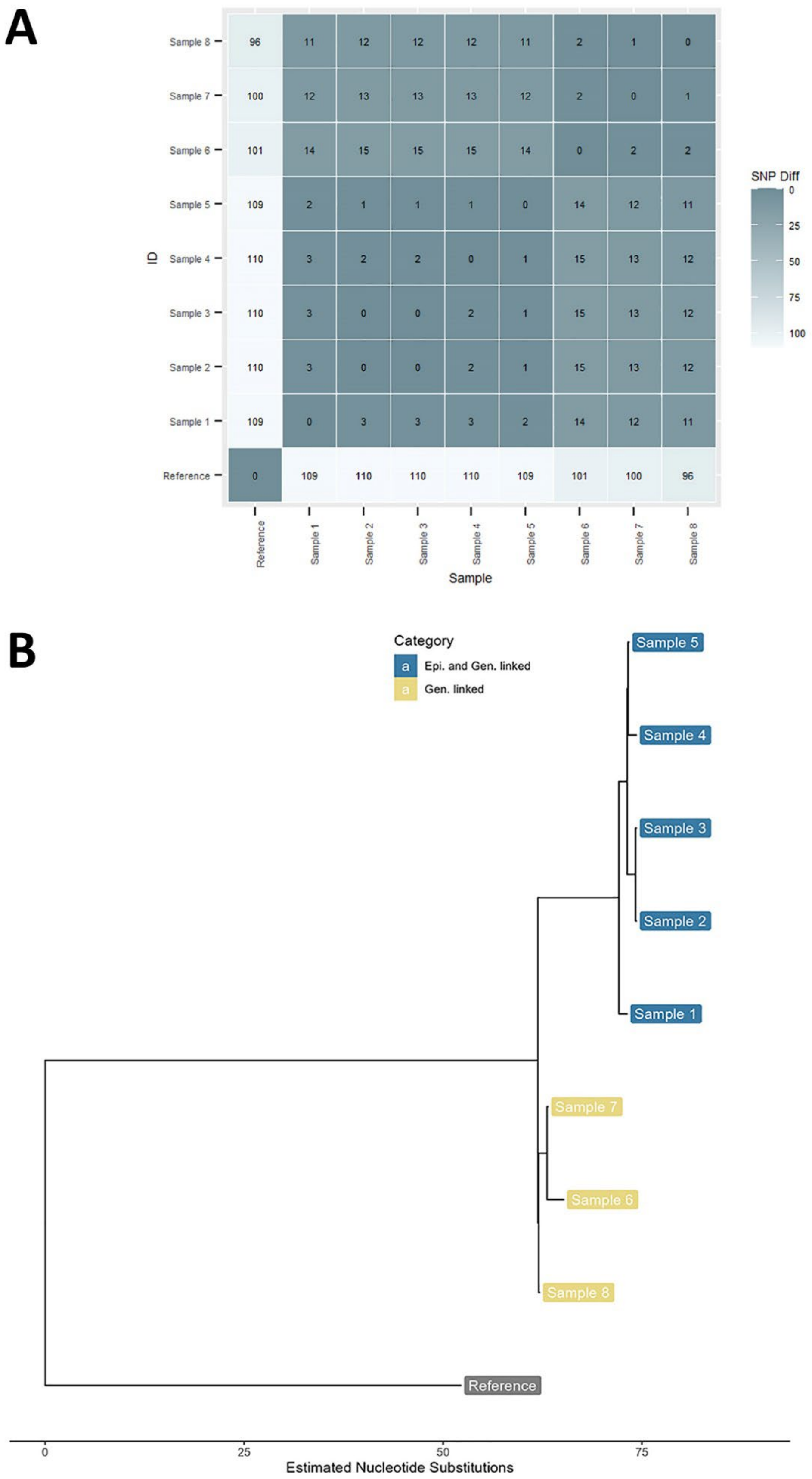
A
SNP Diff
0
25
50
75
100
ID
Sample
Reference
Sample 1
Sample 2
Sample 3
Sample 4
Sample 5
Sample 6
Sample 7
Sample 8
B
Category
Epi. and Gen. linked
Gen. linked
Estimated Nucleotide Substitutions

Appendix 1 Figure 5. A) *A. baumannii* SNP matrix and (B) maximum-likelihood phylogenetic tree summarize genomic and epidemiologic relationships from Outbreak 5. The SNP matrix and the phylogenetic tree are inferred, labeled, and colored as in Appendix Figure 1. This outbreak involved multiple healthcare facilities. All sequences with known epidemiologic linkages were collected between 2022 and 2023 and differed by 10 or fewer SNPs. The remaining 3 isolates had no known epidemiologic linkages. They were collected in 2019 and diverged by (11–15 SNPs) from the other sequences. They were considered to be genomically linked as the amount of diversity could be explained by the difference in collection dates.

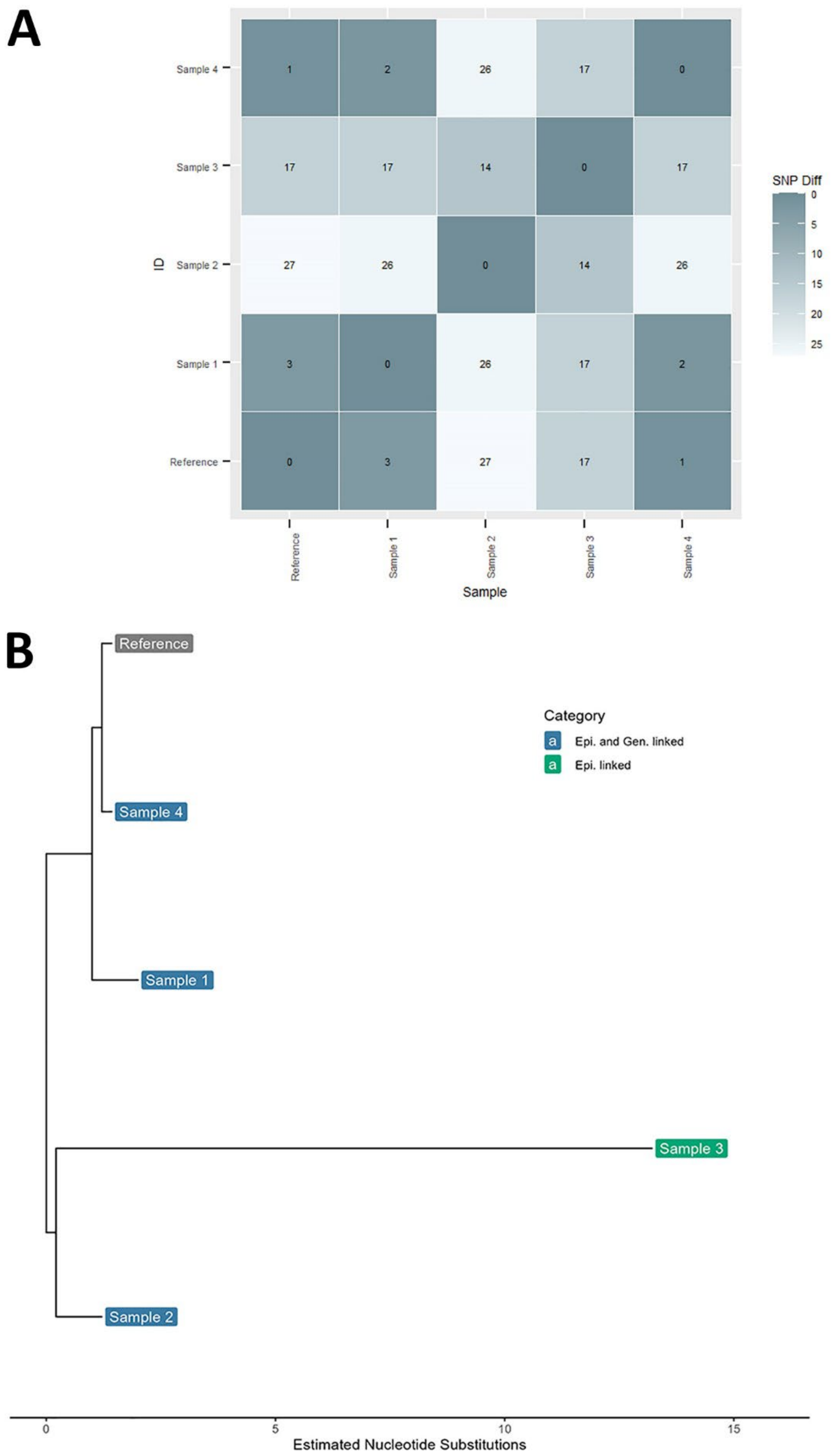
A
SNP Diff
0
5
10
15
20
25
ID
Sample 4
Sample 3
Sample 2
Sample 1
Reference
1
2
26
17
0
17
17
14
0
17
27
26
0
14
26
3
0
26
17
2
0
3
27
17
1
Reference
Sample 1
Sample 2
Sample 3
Sample 4
Sample
B
Reference
Sample 4
Sample 1
Sample 3
Sample 2
Category
Epi. and Gen. linked
Epi. linked
0
5
10
15
Estimated Nucleotide Substitutions

Appendix 1 Figure 6. A) *K. pneumoniae* SNP matrix and (B) maximum-likelihood phylogenetic tree summarize genomic and epidemiologic relationships from Outbreak 6. The SNP matrix and the maximum-likelihood phylogenetic tree are inferred, labeled, and colored as in Appendix Figure 1. Four out of five sequences were epidemiologically and genomically linked (blue tips). Two sequences (Sample 1 and Sample 4) were closely genomically related (diverged by less than 10 SNPs). Sample 2, collected in 2022, diverged by (14–26 SNPs) from the other samples, which were collected in 2023. We considered the time difference between collection dates could account for the observed diversity and categorized this sample as genomically linked. Sample 3, collected in 2023, differed by (14–17 SNPs), was not considered to be genomically linked, as the divergence could not be explained by differences in collection date. This phylogenetic tree does not show a sequence from a case that was epidemiologically linked to this outbreak, but whose sequenced infection was sufficiently diverged that the sample was not included in the cluster. This sequence was found to be genomically linked to a sequence in a different cluster at the same health facility.

Appendix 1 Table 1. Genomic partitions with multiple CRAB isolates with the OXA- like carbapenemase gene in study of genomic and epidemiologic surveillance for multidrug-resistant organisms, Washington, United States*

Genomic partitions, n = 7	No. sequences and cases†	Sequence ID	Epi and genomically linked sequences and cases‡	Genomically linked only sequences and cases§
1	7 sequences from 5 cases	Sample 13<br>Sample 15b, Sample 15c<br>Sample 16a, Sample 16b<br>Sample 17<br>Sample 18	7 sequences from 5 cases<br>Health Facility IV (Cases 13, 15, 16, 17,18)	0
5	3 sequences from 2 cases	Sample 11a, Sample 11b<br>Sample 12	3 sequences from 2 cases<br>Health Facility VII (Cases 11, 12)	0
6	2 sequences from 2 cases	Sample 4<br>Sample 20	0	2 sequences from 2 cases
7	2 sequences from 2 cases	Sample 25<br>Sample 27	0	2 sequences from 2 cases<br>*(Case 25 no epi data, Case 27 no epi data at facilities of interest)*
9	6 sequences from 5 cases	Sample 5<br>Sample 6<br>Sample 7<br>Sample 9a, Sample 9b<br>Sample 10	4 sequences from 4 cases<br>Health Facility I (Cases 5, 7, 10)<br>Health Facility III (Cases 5, 7)<br>Health Facility VIII (Cases 6, 7)	2 sequences from 1 case<br>(Case 9)
10	5 sequences from 4 cases	Sample 1<br>Sample 2<br>Sample 3<br>Sample 8a, Sample 8b	3 sequences from 3 cases<br>Health Facility III (Cases 1, 2, 3)	2 sequences from 1 case<br>(Case 8)
11	3 sequences from 3 cases	Sample 21<br>Sample 23<br>Sample 24	0	3 sequences from 3 cases<br>(*Case 21, 23, and 24 no epi data)*

*There were no sequences identified or cases that were epidemiologically linked only. CRAB, carbapenemase-producing *Acinetobacter baumannii.*
†Total 28 sequences and 23 cases.
‡Total 17 sequences and 14 cases.
§Total 11 sequences and 9 cases.

Appendix 1 Table 2. Genomic partitions with single CRAB isolates with the OXA- like carbapenemase gene epidemiologically linked in study of genomic and epidemiologic surveillance for multidrug-resistant organisms, Washington, United States*

No. sequences and cases	Sequence IDs	Epi linked to cases in facilities
1 sequence from 1 case	Sample 14	Health Facility IV (Cases 13, 15, 16, 17, 18)
1 sequence from 1 case	Sample 15a	Health Facility IV (Cases 14, 16, 17, 18)<br>Health Facility V (Case 18)
1 sequence from 1 case	Sample 19	Health Facility IV (Cases 16, 17)

*Data shown for 3 sequences and 3 cases. CRAB, carbapenemase-producing *Acinetobacter baumannii.*

Appendix 1 Table 3. SRA accession numbers for genome sequences analyzed in study of genomic and epidemiologic surveillance for multidrug-resistant organisms, Washington, United States*

Outbreak	Sample	Spp.	Bio sample	SRA
1	1	Pseudomonas aeruginosa	SAMN33190827	SRR23362665
1	2	Pseudomonas aeruginosa	SAMN33190960	SRR23362574
1	3	Pseudomonas aeruginosa	SAMN36342650	SRR25177959
1	4	Pseudomonas aeruginosa	SAMN34174923	SRR24163236
1	5	Pseudomonas aeruginosa	SAMN42837460	SRR30006055
1	6	Pseudomonas aeruginosa	SAMN36342678	SRR25177928
1	7	Pseudomonas aeruginosa	SAMN37798030	SRR26371322
1	8	Pseudomonas aeruginosa	SAMN37798098	SRR26371360
2	1	Acinetobacter baumannii	SAMN37875388	SRR26424133
2	2	Acinetobacter baumannii	SAMN37875390	SRR26424121
2	3	Acinetobacter baumannii	SAMN37875391	SRR26424110
2	4	Acinetobacter baumannii	SAMN37875392	SRR26424099
2	5	Acinetobacter baumannii	SAMN37875450	SRR26424074
2	6	Acinetobacter baumannii	SAMN39244249	SRR27411051
3	1	Acinetobacter baumannii	SAMN33193792	SRR23652639
3	2	Acinetobacter baumannii	SAMN42837462	SRR30006053
3	3	Acinetobacter baumannii	SAMN42837461	SRR30006054
3	4	Acinetobacter baumannii	SAMN37875424	SRR26424103
3	5	Acinetobacter baumannii	SAMN37875393	SRR26424088
3	6	Acinetobacter baumannii	SAMN31388831	SRR21985580
4	1	Acinetobacter baumannii	SAMN33193741	SRR23652608
4	2	Acinetobacter baumannii	SAMN35130721	SRR24635255
4	3	Acinetobacter baumannii	SAMN34403834	SRR24324145
4	4	Acinetobacter baumannii	SAMN37798018	SRR26371296
4	5	Acinetobacter baumannii	SAMN34410783	SRR24326815
4	6	Acinetobacter baumannii	SAMN36342659	SRR25177949
4	7	Acinetobacter baumannii	SAMN36342638	SRR25177972
4	8	Acinetobacter baumannii	SAMN37798029	SRR26371323
4	9	Acinetobacter baumannii	SAMN34403835	SRR24324144
4	10	Acinetobacter baumannii	SAMN37798044	SRR26371306
5	1	Acinetobacter baumannii	SAMN34174908	SRR24163157
5	2	Acinetobacter baumannii	SAMN34403785	SRR24324051
5	3	Acinetobacter baumannii	SAMN36342649	SRR25177960
5	4	Acinetobacter baumannii	SAMN34403800	SRR24324035
5	5	Acinetobacter baumannii	SAMN35130692	SRR24635287
5	6	Acinetobacter baumannii	SAMN38050710	SRR26617443
5	7	Acinetobacter baumannii	SAMN38050711	SRR26617442
5	8	Acinetobacter baumannii	SAMN42826255	SRR30040684
6	1	Klebsiella pneumoniae	SAMN36342695	SRR25177909
6	2	Klebsiella pneumoniae	SAMN36342697	SRR25177907
6	3	Klebsiella pneumoniae	SAMN37798049	SRR26371301
6	4	Klebsiella pneumoniae	SAMN37798048	SRR26371302
Multi-facility outbreak	PT A	Klebsiella pneumoniae	SAMN36342696	SRR25177908
Multi-facility outbreak	PT B	Klebsiella pneumoniae	SAMN24300518	SRR17311738
Multi-facility outbreak	PT C	Klebsiella pneumoniae	SAMN39244290	SRR27411025
Multi-facility outbreak	PT D	Klebsiella pneumoniae	SAMN39484725	SRR27606182
Multi-facility outbreak	PT E	Klebsiella pneumoniae	SAMN34403744	SRR24324096
Multi-facility outbreak	PT F	Klebsiella pneumoniae	SAMN34403745	SRR24324095
CRAB OXA-235	1	Acinetobacter baumannii	SAMN23011930	SRR16893805
CRAB OXA-235	2	Acinetobacter baumannii	SAMN23011931	SRR16893803

Outbreak	Sample	Spp.	Bio sample	SRA
CRAB OXA-235	3	Acinetobacter baumannii	SAMN33190834	SRR23362614
CRAB OXA-235	4	Acinetobacter baumannii	SAMN31388767	SRR21985548
CRAB OXA-235	5	Acinetobacter baumannii	SAMN37875424	SRR26424103
CRAB OXA-235	6	Acinetobacter baumannii	SAMN33193792	SRR23652639
CRAB OXA-235	7	Acinetobacter baumannii	SAMN31388831	SRR21985580
CRAB OXA-235	8a	Acinetobacter baumannii	SAMN39484729	SRR27606178
CRAB OXA-235	8b	Acinetobacter baumannii	SAMN39484728	SRR27606179
CRAB OXA-235	9a	Acinetobacter baumannii	SAMN42837461	SRR30006054
CRAB OXA-235	9b	Acinetobacter baumannii	SAMN42837462	SRR30006053
CRAB OXA-235	10	Acinetobacter baumannii	SAMN37875393	SRR26424088
CRAB OXA-235	11b	Acinetobacter baumannii	SAMN31388819	SRR21985490
CRAB OXA-235	11a	Acinetobacter baumannii	SAMN37875398	SRR26424131
CRAB OXA-235	12	Acinetobacter baumannii	SAMN33190870	SRR23362527
CRAB OXA-235	13	Acinetobacter baumannii	SAMN33193741	SRR23652608
CRAB OXA-235	14	Acinetobacter baumannii	SAMN34403834	SRR24324145
CRAB OXA-235	15c	Acinetobacter baumannii	SAMN37798029	SRR26371323
CRAB OXA-235	15a	Acinetobacter baumannii	SAMN34403835	SRR24324144
CRAB OXA-235	15b	Acinetobacter baumannii	SAMN34410783	SRR24326815
CRAB OXA-235	16a	Acinetobacter baumannii	SAMN36342659	SRR25177949
CRAB OXA-235	16b	Acinetobacter baumannii	SAMN36342638	SRR25177972
CRAB OXA-235	17	Acinetobacter baumannii	SAMN37798044	SRR26371306
CRAB OXA-235	18	Acinetobacter baumannii	SAMN35130721	SRR24635255
CRAB OXA-235	19	Acinetobacter baumannii	SAMN37798018	SRR26371296
CRAB OXA-235	20	Acinetobacter baumannii	SAMN31407601	SRR22007350
CRAB OXA-235	21	Acinetobacter baumannii	SAMN23011920	SRR16893816
CRAB OXA-235	22	Acinetobacter baumannii	SAMN23011929	SRR16893806
CRAB OXA-235	23	Acinetobacter baumannii	SAMN23011909	SRR16893777
CRAB OXA-235	24	Acinetobacter baumannii	SAMN23011908	SRR16893778
CRAB OXA-235	25	Acinetobacter baumannii	SAMN23011884	SRR16913122
CRAB OXA-235	26	Acinetobacter baumannii	SAMN35130650	SRR24635284
CRAB OXA-235	27	Acinetobacter baumannii	SAMN23011912	SRR16893824

* CRAB, carbapenemase-producing *Acinetobacter baumannii;* PT, patient.
