# Supplementary material for: Integrating Genomic Data into Public Health Surveillance for Multidrug-Resistant Organisms, Washington, USA
Source: Emerg Infect Dis. 2025 May;31(Suppl 1):S25–34. doi: 10.3201/eid3113.241227 (PMC12078550; doi:10.3201/eid3113.241227)
Supplement: Appendix 2 — Example of automated summary report in study of integrating genomic data into public health surveillance for multidrug-resistant organisms, Washington, USA. [file 24-1227-Techapp-s2.pdf]

Article DOI: https://doi.org/10.3201/eid3113.241227

EID cannot ensure accessibility for supplementary materials supplied by authors. Readers who have difficulty accessing supplementary content should contact the authors for assistance.

Integrating Genomic Data into Public Health Surveillance for Multidrug-Resistant Organisms, Washington, United States

Appendix 2

Report Sample

The following pages show an example of our automated summary report with mock-up data (https://github.com/NW-PaGe/BacterialGenomicsSummaryOutput). The report contains tables that summarize key details about the genomic clusters identified through BigBacter (https://github.com/DOH-JDJ0303/bigbacter-nf) such as the total number of sequences in each cluster, number of new sequences added to previously identified clusters, names of submitting health facilities and submitting counties, and which sequences show evidence of close linkage. The report is shared along with genomic interpretations and Microreact files.

Bacterial Genomics - Summary Report

AUTHOR
Molecular Epidemiology Program, WA DOH

PUBLISHED
November 22, 2024

Overview New Samples

There are 2 new sequencing results in this BigBacter run.
This is the mapping of the sequencing ID to the corresponding case ID.

ID	WA_ID	CASE_ID
**Sample c**	WA0000000	100000000
**Sample e**	WA9999999	109999999

The new isolates were classified by BigBacter as follows:

ID	QUAL	TAXA	GENOMIC CLUSTER	ISO IN CLUSTER	ISO PASS QC
**Sample c**	PASS	Klebsiella_pneumoniae	8	6	6
**Sample e**	PASS	Pseudomonas_aeruginosa	17	1	1

Of those, the following isolates resulted in new genetic clusters.

ID	QUAL	TAXA	GENOMIC CLUSTER	ISO IN CLUSTER	ISO PASS QC
**Sample e**	PASS	Pseudomonas_aeruginosa	17	1	1

Failed Isolates

The following isolates failed quality control.

There weren't any isolates that failed quality control in this run

Recombination

Bacterial recombination is the process where bacteria exchange genetic material with each other which leads to the gain of new DNA sequences into their genomes. It is important to be aware of recombination when conducting genomic analyses because recombination events can be confused with mutations events which can impact metrics used to characterize relationships between sequences, such as calculating single nucleotide polymorphisms (SNP) distances. The bioinformatics pipelines developed at WA PHL use Gubbins, a method to detect and control for recombination. If recombination is detected the sites where recombination is present are masked in the SNPs distance calculations and in the phylogenetic trees.
We evaluate recombination in multiple ways. First the number of sites where recombination was detected is divided by the total length of the core genome. If recombination is more than 5% in a genomic cluster the Gubbins outputs are used. If recombination is more than 1% but less than 5%, then the Snippy and Gubbins outputs are reviewed jointly to see if they yield different interpretations. If the interpretations differ, then most likely we will use the Gubbins for the genomic interpretations.

TAXA	GENOMIC CLUSTER	MAX_%Recomb_Detected
**Klebsiella_pneumoniae**	8	4.373

Sequences that resulted in new genetic clusters are excluded from this calculation.

SNP Min and Max Distances

The minimum and maximum SNP distances for each genomic cluster by each method, Snippy and Gubbins, are summarized below

Source	MAX	MIN
**Klebsiella_pneumoniae-00008-core-snps_dist.gubbins-long**	381	2

**Klebsiella_pneumoniae-00008-core-snps_dist.snippy-long**	2,454	3

Sequences that resulted in new genetic clusters are excluded from this calculation.

Genomic Linkages

Based on the SNP distances these are the strong (0-10 SNPs) and intermediate (11-50 SNPs) linkages found among the new isolate/s and other sequences in the corresponding genomic clusters.

Klebsiella_pneumoniae-00008-core-snps_dist.gubbins-long

ID	Strong Genetic Linkages (0-10 SNPs)	Intermediate Genetic Linkages (11-50 SNPs)
**Sample c**	Sample a	
**Sample c**	Sample b	

Klebsiella_pneumoniae-00008-core-snps_dist.snippy-long

ID	Strong Genetic Linkages (0-10 SNPs)	Intermediate Genetic Linkages (11-50 SNPs)
**Sample c**	Sample a	
**Sample c**		Sample b

Metadata

This is an overview of the metadata pertaining to each of the genomic clusters that contain new isolates. The facilities are the submitting facilities and the counties the submitting facilities' county.

Taxa_Genomic Cluster	Min Collection date	Max Collection date	All Counties	New Counties	All Facilities	New Facilities	All IDs	New IDs	ISOs Same Case
**K_pneumoniae_8**	10-16-22	07-11-24	County x	County x<br>County z	Facility I<br>Facility II<br>Facility III	Facility III	a, c, f, g, h, i	c	DOB: 1-1-1999<br>g, h
**P_aeruginosa_17**	09-10-24	09-10-24	County x	County x	Facility II	Facility II	e	e	No isolates from the same case

Resources

The code to generate this report is available here:
https://github.com/NW-PaGe/BacterialGenomicsSummaryOutput
The following bioinformatics methods were used by WA PHL to generate some of the data summarized in this report.
BigBacter bioinformatics pipeline https://github.com/doh-jdj0303/bigbacter-nf
Snippy https://github.com/tseemann/snippy
Gubbins https://github.com/nickjcroucher/gubbins
